# Among the Colleges

**Published:** 1899-08

**Authors:** 


### AMONG THE COLLEGES.

THE ninth annual announcement of the Kansas City Veterinary College has been received, in which the following changes are noted: The number of directors of the college have been reduced to seven, dropping from the list Silas L. Brooking, M.D., D.V.S., of Olathe, Kansas. Changes in the Faculty: Robert C. Moore, D.V.S., has taken the place of Charles J. Sihler, V.S., as professor of anatomy; James H. Cock, D.V.S., has also been added as professor of anatomy; Alfred H. Rogers, A.M., M.D., has been added as one of the professors of physiology; W. Ross Cooper, D.V.M., has taken the place of Wm. C. Barth, V.S., and R. P. Steddom, V.S., as professor of obstetrics. The branch of chemistry has been divided into two parts: Alphonso M. Hunt, M.D., as professor of inorganic chemistry, and Theodore Schaefer, M.D., as professor of organic chemistry. Wm. A. Nixon, V.S., has taken the place of R. H. Harrison, D.V.S., as professor of veterinary dentistry. James Otterman is added as professor of materia medica and therapeutics in place of Richard J. Blanch, D.V.M. James H. Cock dropped as demonstrator of materia medica and therapeutics. Albert Long, M.D.V., professor of hygiene, in place of E. von Quast, M.D., and professor of control and eradication of contagious diseases, in place of George A. Johnson, D.V.M. Frank C. McCurdy, V.M.D., in place of J. C. Milnes, V.S., as professor of canine pathology. Joseph S. Brooks, A.B., B.L., added as professor of veterinary jurisprudence, in place of Frank M. Sheridan, Esq. Charles Saunders, D.V.S., added as professor of pathological shoeing, in place of T. A. Bray, D.V.S., on lameness and shoeing. Demonstrator of anatomy, formerly filled by Don C. Patten, D.V.M., remains unfilled. All other branches are as last year.

*Materia Medica and Therapeutics*, by H. D. Hanson and E. L. Quitman, and *Shoeing*, by Dollar, have been added as reference books. Dropped from the list: *Histology*, by Gottheil, and *Dictionary*, by Thomas and Billings.

The following graduates received the degree of D.V.S., March, 1899: Nelson V. Boyce, Kansas City, Kansas; Henry G. Patterson, St. Joseph, Missouri; Harold C. Simpson, Denison, Iowa; Charles E. Steel, North Topeka, Kansas.

